# Diazoxide Causality Assessment of a Pericardial Effusion in a Child with Kabuki Syndrome

**DOI:** 10.4274/jcrpe.galenos.2018.2018.0193

**Published:** 2019-05-28

**Authors:** Irene Maffre, Marie Vincenti, Fabienne Dalla Vale, Cyril Amouroux, Oscar Werner, Alexandra Meilhac, Gaelle de Barry, Pascal Amedro

**Affiliations:** 1 M3C Regional Centre, University Hospital, Department of Pediatric and Congenital Cardiology, Montpellier, France; 2 University Hospital, Department of Hospital Pharmacy, Montpellier, France; 3 University of Montpellier, PhyMedExp, CNRS, INSERM, Montpellier, France; 4 M3C Regional Centre, University Hospital, Department of Pediatric and Endocrinology Nephrology, Montpellier, France

**Keywords:** Pericardial effusion, diazoxide, Kabuki syndrome, paediatrics

## To the Editor,

A 15 month-old girl with KS (KDM6A mutation) was referred to our tertiary care paediatric cardiology centre for respiratory and hemodynamic distress. Her medical history involved congenital hypothyroidism treated from birth by levothyroxine in addition to congenital hyperinsulinism and renal malformations. She had been treated by diazoxide for 10 months in a dose of 10 mg/kg/d with a 5% maltose dextrin diet, under correct glycaemic monitoring. The pediatric cardiologist confirmed by echocardiography the absence of any congenital heart disease (CHD) or pulmonary hypertension (PH), but diagnosed a severe pericardial effusion. Ibuprofen and colchicine were started, successively, but had no effect. On day 12, a pericardial puncture was performed. The results of routine etiological assessment for pericarditis (brain natriuretic peptide, troponin, thyroid status, and common viruses) were negative. On day 16, the diazoxide was suspended and a continuous diet with 10% maltose dextrin was introduced. The pericardial effusion started to regress on day 25 and disappeared on day 28. On day 29, in order to preserve the patient’s pancreatic function, diazoxide was reintroduced in a low dose (3 mg/kg/d) under close echocardiographic monitoring. On day 34 (5 days after diazoxide was reintroduced), a 3-mm pericardial effusion was diagnosed. The diazoxide was then increased to 4.5 mg/kg/d and food intake was decreased to 5% maltose dextrin. On day 36, the pericardial effusion was noted to be stable (3 mm). However, on day 47, e.g. 19 days after reintroduction of diazoxide, pericardial effusion significantly increased to 6 mm. Therefore, diazoxide was definitively stopped. On day 52, e.g. five days after diazoxide was stopped, the echocardiography showed a regression of the pericardial effusion. Currently, after a six-month follow-up since diazoxide was suspended, echocardiography assessments have revealed normal results.

We report a new case with an adverse event of diazoxide. This patient, a 15-month-old girl with Kabuki syndrome (KS), developed a severe pericardial effusion following diazoxide. We performed, for the first time, a causality assessment of this drug toxicity and found a high probability of a causal relationship between diazoxide and pericardial effusion. Indeed, after the treatment was suspended, the pericardial effusion regressed and reappeared when diazoxide was reintroduced. Using the Naranjo algorithm, the adverse drug reaction probability scale total score was rated at 10 (e.g. the reaction is considered definite if the score is 9 or higher) (1).

KS is a rare multiple congenital malformation syndrome, in which CHD have been described. However, pericardial effusion is not a common complication. Two cases of potential toxicity of diazoxide with pericardial effusion have been previously reported (2,3). A recent retrospective study on 295 children with congenital hyperinsulinism reported that 2.4% were diagnosed with PH after diazoxide initiation (4). Indeed, pericardial effusion may occur in severe PH, however, but our patient did not present with PH. Another drug with a similar action, the minoxidil, was suspected to be associated with pericardial effusion, that may be explain the cardiac toxicity of the drug (5).

We recommend monitoring patients with KS under diazoxide with echocardiography to detect pericardial effusion.
